# Supplementary material for: Fabrication of Silk Fibroin Fluorescent Nanofibers via Electrospinning
Source: Polymers (Basel). 2019 Jun 4;11(6):986. doi: 10.3390/polym11060986 (PMC6631164; doi:10.3390/polym11060986)
Supplement: Supplementary file 1 [file polymers-11-00986-s001.pdf]

Supplementary information

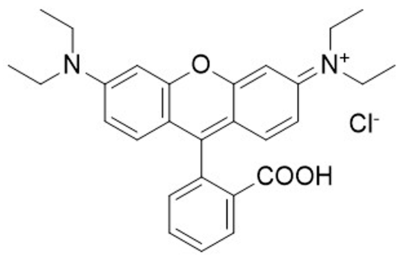

Figure S1 Chemical formula of rhodamine B (RB)

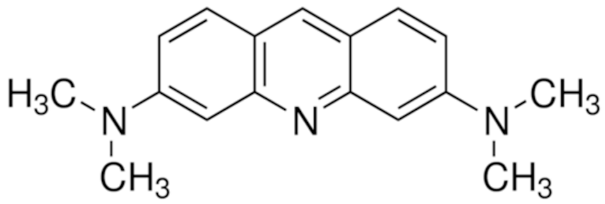

Figure S2 Chemical formula of acridine orange (AO)

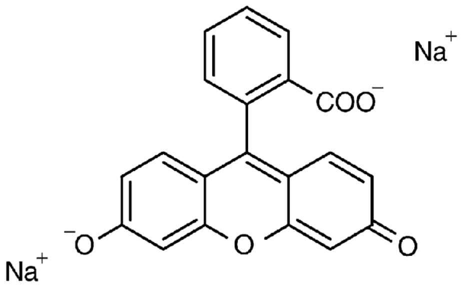

Figure S3 Chemical formula of fluorescein sodium (FS)

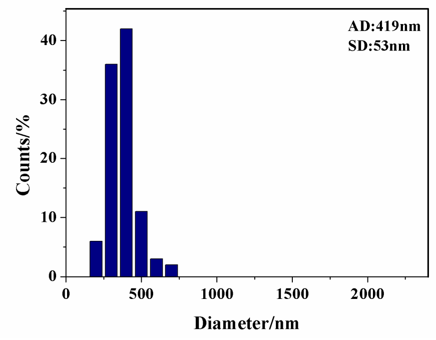

(a)

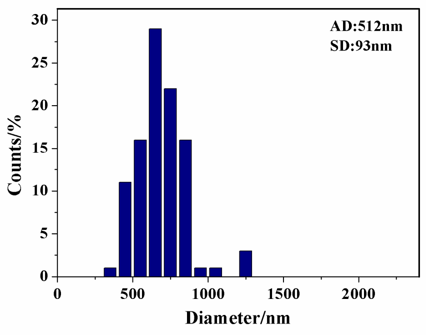

(b)

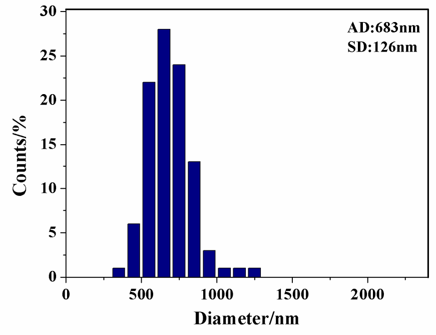

(c)

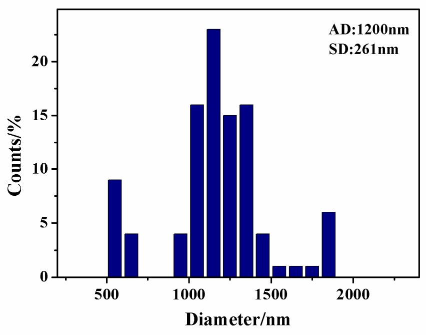

(d)

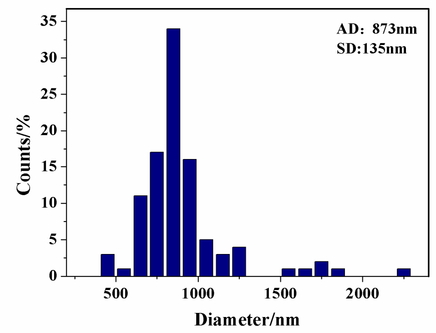

(e)

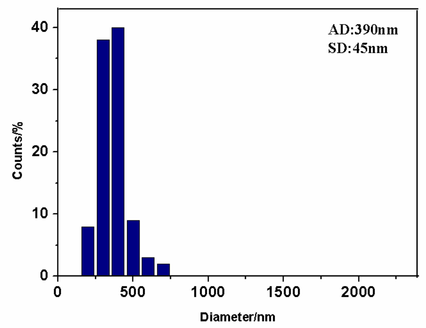

(f)

Figure S4 SEM images of diameter distributing of fluorescent SF-FS nanofibers with different SF and FS concentration. The concentration of SF was (a) 6.0 wt%, (b) 8.0 wt%, and (c,d,e) 10.0 wt%. The concentration of FS was (a,b,c) 1.0 wt%, (d) 0.5 wt%, and (e) 2.0 wt%, respectively. (f) pure SF nanofibers with 6.0 wt% SF concentration.

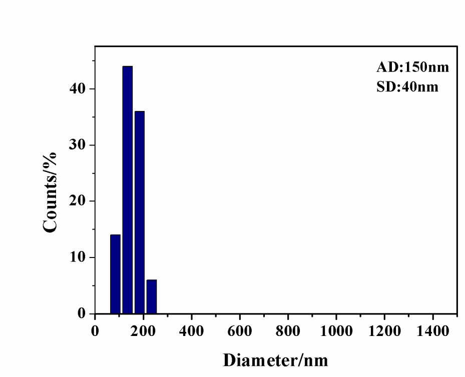

(a)

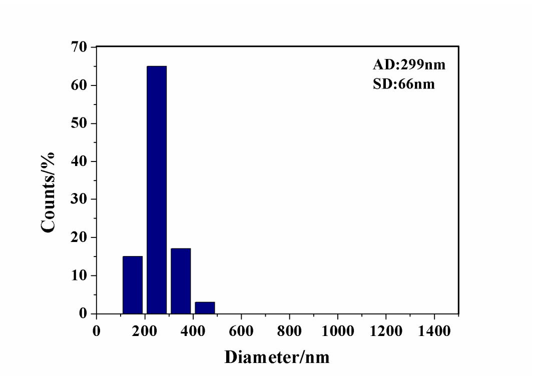

(b)

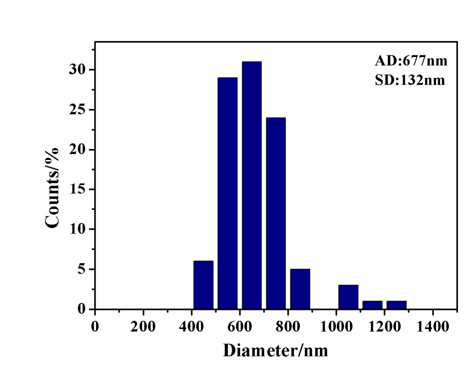

(c)

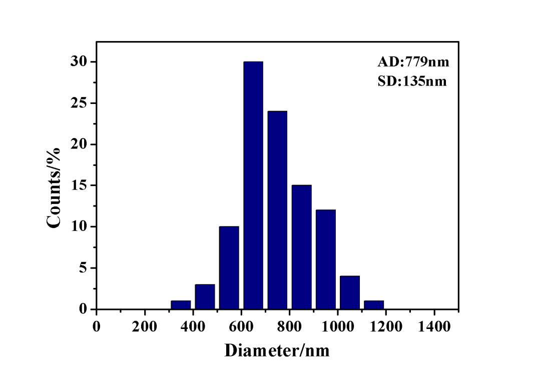

(d)

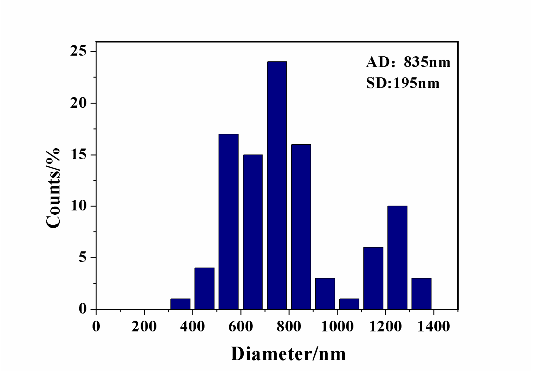

(e)

Figure S5 SEM images of diameter distributing of fluorescent SF-RB nanofibers with different SF and RB concentration. The concentration of SF was (a) 6.0 wt%, (b) 8.0 wt%, (c,d,e) 10.0 wt%. The concentration of RB was (a,b,c) 1.0 wt%, (d) 0.5 wt%, and (e) 2.0 wt%, respectively.

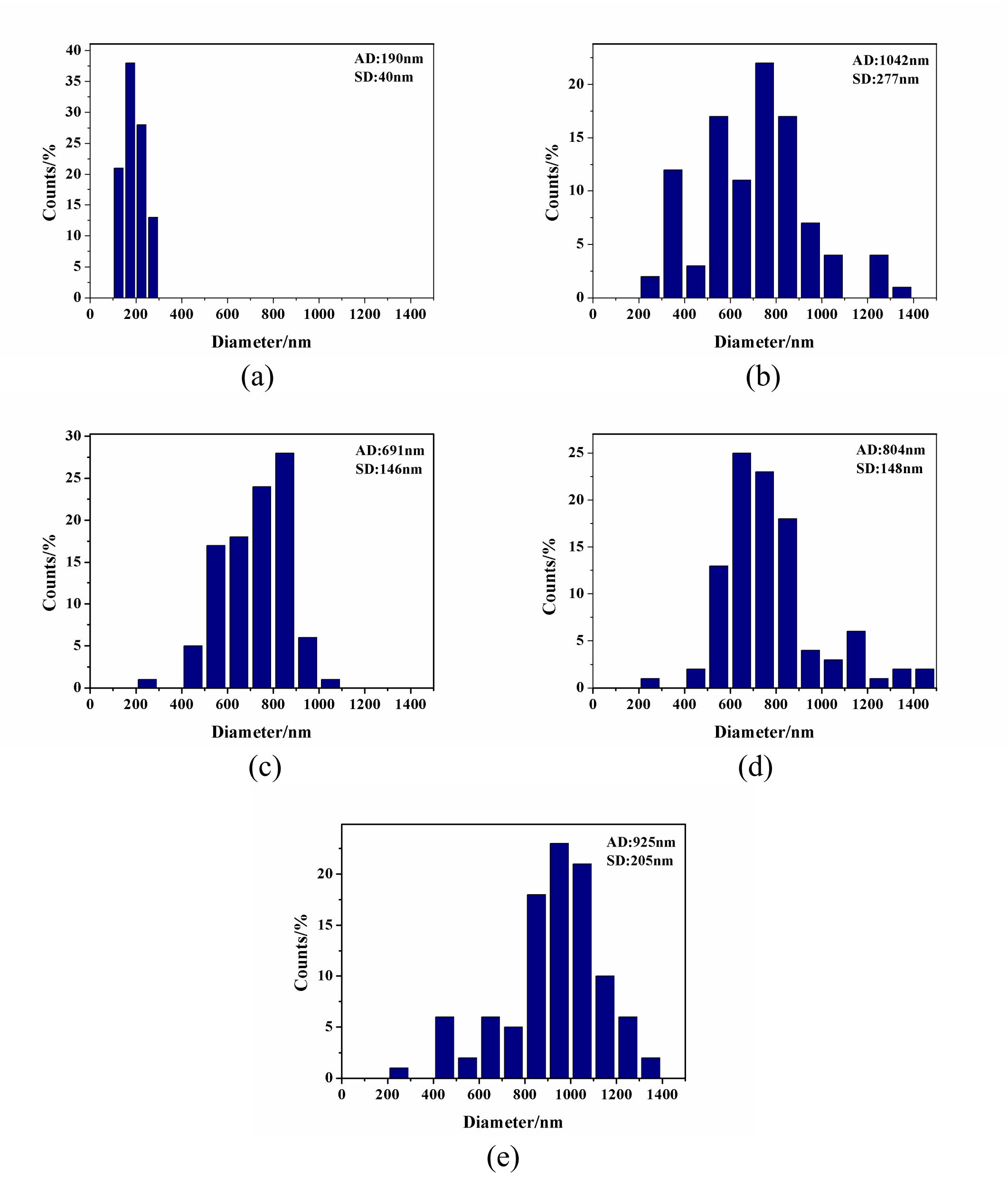

Figure S6 SEM images of diameter distributing of fluorescent SF-AO nanofibers with different SF and AO concentration. The concentration of SF was (a) 6.0 wt%, (b) 8.0 wt%, (c,d,e) 10.0 wt%. The concentration of AO was (a,b,c) 1.0 wt%, (d) 0.5 wt%, and (e) 2.0 wt%, respectively.
